# The Promoting Effect of Reactive Comb Compatibilizer on the Formation of Co-Continuous Structure in PVDF/PLLA Blends

**DOI:** 10.3390/polym18131586

**Published:** 2026-06-26

**Authors:** Yufei Dong, Fei Li, Jiayao Wang, Yongjin Li, Guipeng Yu, Jichun You

**Affiliations:** 1 College of Chemistry and Chemical Engineering, Central South University, Changsha 410083, China; 20243007@hznu.edu.cn; 2 Key Laboratory of Organosilicon Chemistry and Material Technology, College of Material, Chemistry and Chemical Engineering, Zhejiang Key Laboratory of Organosilicon Material Technology, Ministry of Education, Hangzhou Normal University, Hangzhou 311121, Chinajiayaowang@hznu.edu.cn (J.W.); yongjin-li@hznu.edu.cn (Y.L.)

**Keywords:** reactive compatibilizer (RC), co-continuity, phase diagram, viscosity ratio, phase coalescence

## Abstract

While many kinds of reactive compatibilizers (RCs) have been artificially created for reducing interfacial tension in immiscible polymer blends, the effect of RCs on co-continuity formation (preferred in application) remains controversial. In this work, we selected the previously reported poly(vinylene fluoride)/poly(L-lactic acid)/reactive comb compatibilizer (PVDF/PLLA/RCC) blend system as the model system, in which the reaction between epoxy groups in RCC and terminal carboxyl groups in PLLA upon blending can produce glycidyl methacrylate (GMA)-PLLA side chains located at the PVDF/PLLA interface. By manipulating PVDF/PLLA composition ratio and RCC content, a half-U-shaped co-continuous phase diagram with an upward opening was obtained, suggesting a promoting effect of RCC on PVDF/PLLA co-continuity. This can be attributed to both the high viscosity of PLLA/RCC and the inhibited PVDF phase coalescence. On the one hand, owing to the significantly increased viscosity of PLLA/RCC relative to neat PLLA, the decreased viscosity ratio of PVDF to PLLA(/RCC) can lower the co-continuous PVDF/PLLA composition ratio. On the other hand, the coalescence of PVDF phase in PVDF/PLLA/20%RCC can be effectively inhibited due to the addition of RCC, opposite to the inclined PLLA phase coalescence in the neat PVDF/PLLA blend with high PVDF fraction (e.g., 80%). Our results provide guidance for evaluating and selecting RCs for a specific immiscible polymer blend in practical application.

## 1. Introduction

Polymer blending is a simple and effective way of developing high-performance materials with combined advantages of the constituents [1,2,3]. The microstructure of a polymer blend is highly dependent on the interfacial interaction (compatibility) between the polymers. Miscible polymer blends [e.g., poly(vinylidene fluoride) (PVDF)/poly(methyl methacrylate) (PMMA) [4,5], high-density polyethylene (HDPE)/styrene-ethylene-butylene (SEB) [6], polycarbonate (PC)/PMMA [7]] with strong interfacial interaction can exhibit homogeneous structures without visible microphase separation. By contrast, for immiscible polymer blends with inferior interfacial adhesion between the constituents [e.g., HDPE/PS [6], PS/polyamide 6 (PA6) [8], PC/polypropylene (PP) [9]], phase separation structures (typically sea-island or co-continuous) are always presented. Compared with sea-island structures, co-continuous ones with improved homogeneity and continuity can endow immiscible polymer blends with more stress/charge/heat/sound transfer channels and are therefore more favorable in application [10,11,12].

Block/graft co-polymers [6,13,14] or reactive compatibilizers (RCs) [15,16,17] have been widely used to decrease the interfacial tension between immiscible polymers and suppress phase coalescence, with the aid of the reactive groups within RCs that can react with their complementary groups in at least one constituent of polymer blends. However, conflicting conclusions have long existed regarding the influence of RCs on the evolution of microphase structure during polymer blending. Some works [18,19] proposed that the addition of RCs is negative for co-continuity formation in polymer blends. For instance, the co-continuity intervals (defined as the range of blend compositions with nearly 100% co-continuity) of poly(lactic acid) (PLA)/PS blend compatibilized by 1% vinyl oxazoline were reported to be narrower than that of the non-compatibilized one (20% vs. 40%) [18]. By contrast, some other works [20,21] reported a promoting effect of RCs on co-continuity formation. The typical example is the PVDF/poly(L-lactic acid) (PLLA) blend system [20], whose co-continuity interval is wider in the blend with 1 wt% poly(styrene-co-glycidyl methacrylate)-graft-poly(methyl methacrylate) (RC-SG) relative to the one without compatibilizer (from 0% to 25%). Given the current situation and complex factors influencing the structural evolution during polymer blending, e.g., polymer viscosity [22,23,24], blending speed and time [25,26], building phase diagram relevant to both constitute composition and RC content at fixed blending conditions can be the simplest way of illustrating the role of RC addition for a specific polymer blend system.

In this work, therefore, we took the PVDF/PLLA/reactive comb compatibilizer (RCC) blend system reported in our previous works [27,28,29,30,31] as the model system, in which the RCC with epoxy groups could react with PLLA rather than PVDF. Then, we investigated the influence law of RCC on its microphase structure by determining the opening direction of the co-continuous phase diagram (facing up or down). Based on both the scanning electron microscopy (SEM) morphology and acidolysis rate of PVDF/PLLA/RCC blends with various compositions, a half-U-shaped phase diagram with upward opening was ultimately presented, suggesting a promoting effect of RCC on co-continuity formation. The co-continuity interval was ultimately expanded from 10% (60/40 to 70/30) for a neat PVDF/PLLA blend to a wide range of ~50% (10/90 to 60/40) for a PVDF/PLLA/20%RCC blend, mainly attributed to the dramatically increased viscosity of PLLA/RCC (relative to PLLA) and the inhibition effect of RCC on PVDF phase coalescence.

## 2. Experimental Section

### 2.1. Materials

PVDF (KF850, M_w_ = 209,000 g/mol, PDI = 2.0) and PLLA (3001D, M_w_ = 89,300 g/mol, PDI = 1.8) were purchased from Kureha Chemicals (Tokyo, Japan) and Nature Works (Blair, NE, USA), respectively. The synthesis and parameters of reactive comb compatibilizer (RCC, C-1-1-8 S-2400, M_w_ = 24,000 g/mol, PDI = 2.1) have been described in Ref. [30]. The RCC is composed of poly(methyl methacrylate) (PMMA) backbone with randomly distributed PMMA side chains as well as glycidyl methacrylate (GMA) chains with reactive epoxy groups. Concentrated nitric acid (analytical grade) was purchased from Zhejiang Zhongxing Chemical Reagent Co., Ltd. (Lanxi, China).

### 2.2. Preparation of PVDF/PLLA/RCC Samples

PVDF, PLLA, and RCC were dried in a vacuum oven at 80 °C overnight in order to remove moisture before melt blending. All the PVDF/PLLA/RCC blends were prepared melt mixing in a batch mixer (Haake Polylab QC, Thermo Fisher Scientific Inc., Waltham, MA, USA) at 190 °C, with a 20 rpm rotation for 2 min and a following 50 rpm rotation for 10 min. The weight ratio of PVDF/PLLA was settled in a range of 0/100 to 100/0, with an addition of 0 wt%, 3 wt%, 5 wt%, 10 wt%, and 20 wt% RCC. Upon mixing, PLLA chain extension is induced by the grafting of a portion of PLLA chains onto the GMA side chains within RCC, without any signal for PLLA degradation (Figure S1). The PLLA grafting ratios of the blends with 3 wt%, 5 wt%, 10 wt%, and 20 wt% RCC were determined as ~18%, ~50%, ~65%, and ~70% respectively, according to the method described in Ref. [28]. Then, the blends were compression-molded under 10 MPa at 200 °C, in order to obtain 10 × 10 cm^2^ PVDF/PLLA/RCC square sheets with a thickness of 0.5 mm.

### 2.3. Sample Characterization

The microstructures of the PVDF/PLLA/RCC blends were observed by SEM (Hitachi S-4800, Tokyo, Japan) with an accelerating voltage of 3.0 kV. The specimens were fractured in liquid nitrogen and sputter-coated with gold before observation. Acid hydrolysis tests of the samples were performed in a 20% nitric acid aqueous solution at 90 °C until the sample weights remained unchanged. Rheological measurements were conducted with an MCR302 rotational rheometer (Anton Paar, Graz, Styria, Austria) in a small amplitude oscillatory shear (SAOS) frequency sweep mode from 0.01 rad s^−1^ to 500 rad s^−1^, at 5% test amplitude and 230 °C.

## 3. Results

### 3.1. Co-Continuity Interval for PVDF/PLLA/RCC Blends

**Figure 1:**
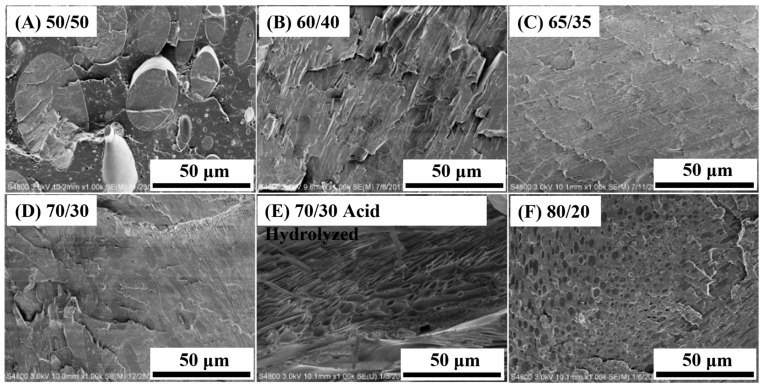
The SEM images of (**A**) 50/50, (**B**) 60/40, (**C**) 65/35, (**D**) 70/30, (**E**) acid-hydrolyzed 70/30, and (**F**) 80/20 poly(vinylene fluoride)/poly(L-lactic acid) (PVDF/PLLA) blends without reactive comb compatibilizer (RCC).

In order to plot the co-continuous phase diagram for the PVDF/PLLA/RCC blend system, the co-continuity interval of the neat PVDF/PLLA blend without RCC was first investigated by SEM. In the blend with a composition of 50/50, an obvious sea-island structure with PVDF (lighter-colored part) as the island phase is presented (Figure 1A). As composition is increased to 60/40 and 65/35, a co-continuous microstructure is formed, without visible phase domains (Figure 1B,C). When the composition is further increased to 70/30, some indeterminate islands are emerged, making it difficult to judge whether the blend is co-continuous or not (Figure 1D). Under the condition, acid hydrolysis was performed on the PVDF/PLLA 70/30 blend so as to selectively degrade the PLLA phase. As a result, the continuity of both PVDF and PLLA phases can be revealed by the acid hydrolysis rate (AHR, defined as mass loss ratio of PLLA in blend specimens [28,29]). An AHR lower than 100% corresponds to a sea-island structure with PLLA islands of continuity equal to the AHR, due to the fact that acid solution hardly contacts the PLLA islands surrounded by PVDF matrix and takes its effect. On the other hand, an AHR significantly higher than 100% implies a reverse structure with PVDF islands of (200—AHR) continuity, owing to the falling of PVDF islands along with the hydrolysis of the surrounding PLLA matrix. Therefore, only AHRs close to 100%, corresponding to a complete degradation of PLLA with fully remaining PVDF, can indicate a co-continuous structure with 100% PLLA and PVDF continuities. The PVDF/PLLA 70/30 blend was ultimately determined as co-continuous, as revealed by the continuous structure (large pores inside can be attributed to the tendency to form a sea-island structure) upon acid hydrolysis (Figure 1E and Figure S2B) along with an AHR of 99.9%. The co-continuous structure of the 70/30 blend was also confirmed by its solvent-extracted morphology in Figure S2A similar to that in Figure S2B as well as the solvent extraction rate of 99.2%. This is based on the fact that PLLA within a co-continuous structure rather than sea-island structures can be extracted by chloroform [28,29,30]. Regarding the PVDF/PLLA 80/20 blend, a sea-island microstructure with PLLA as the island phase is suggested by the emergence of black domains in the light matrix (Figure 1F). Based on the above results, one can tell that the PVDF/PLLA co-continuity interval of the neat PVDF/PLLA blends is 60/40~70/30.

**Figure 2:**
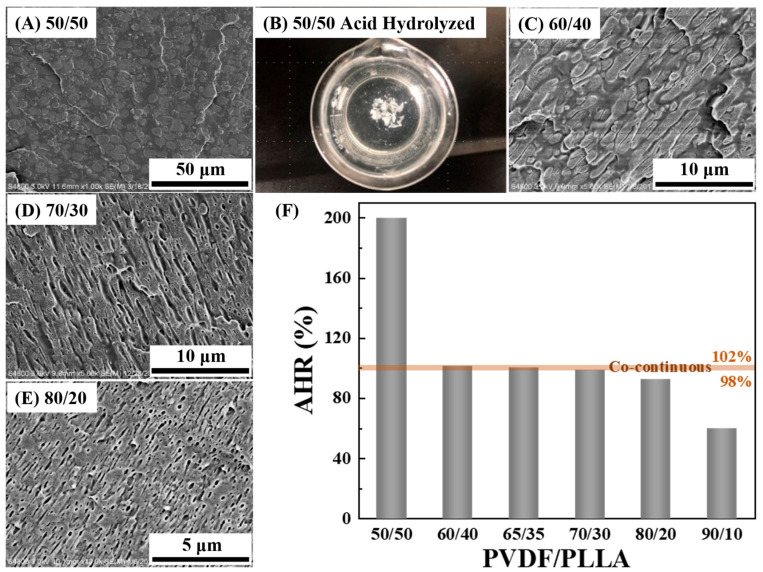
The SEM images of (**A**) 50/50, (**B**) acid-hydrolyzed 50/50, (**C**) 60/40, (**D**) 70/30 and (**E**) 80/20 PVDF/PLLA blends with 3% RCC. (**F**) The acid hydrolysis rates (AHRs) of the PVDF/PLLA/3%RCC blends with various PVDF/PLLA compositions.

The co-continuity of the PVDF/PLLA blend with 3% RCC was then examined. Similar to the neat PVDF/PLLA 50/50 blend (Figure 1A), the PVDF/PLLA/3%RCC 50/50 blend is composed of numerous light PVDF islands dispersed in dark PLLA matrix (Figure 2A), revealing a sea-island microstructure. The sea-island structure of the blend without any PVDF continuity was also confirmed by its crumbled state upon acidolysis (Figure 2B). The microphase structure of the PVDF/PLLA/3%RCC blends with higher PVDF fraction (e.g., 60/40, 70/30, and 80/20) cannot be confirmed merely from their SEM images (Figure 2C–E), and was further checked by acidolysis. According to the AHRs of 101.5%, 100.5% and 99.0% (in the range of 98%~102% close to 100%, Figure 2F) for 60/40, 65/35 and 70/30 blends respectively, their microstructures were confirmed to be co-continuous. By contrast, sea-island microstructures with PLLA islands were implied by lower AHRs of 92.8% and 60% (corresponding to 92.8% and 60% PLLA continuity) for 80/20 and 90/10 blends, respectively (Figure 2F). As a result, the PVDF/PLLA co-continuity interval of the PVDF/PLLA/3%RCC blends is 60/40~70/30, the same as that of the neat PVDF/PLLA blends, which is most likely due to the tiny amount of RCC.

**Figure 3:**
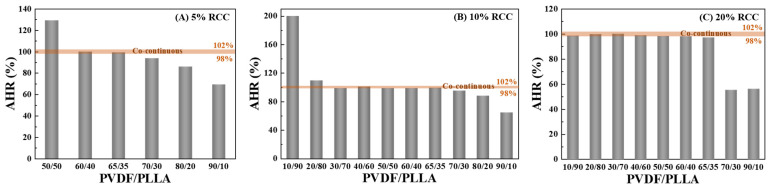
The AHRs of (**A**) PVDF/PLLA/5%RCC, (**B**) PVDF/PLLA/10%RCC, and (**C**) PVDF/PLLA/20%RCC blends with various PVDF/PLLA compositions.

Based on the discussion above, it can be found that acidolysis is more effective in determining the co-continuity of the PVDF/PLLA blends, relative to SEM morphology analysis. Therefore, acid hydrolysis was also performed on the PVDF/PLLA/RCC blends with elevated RCC contents (i.e., 5%, 10%, and 20%) and various PVDF/PLLA compositions. The AHRs of all the blends are presented in Figure 3. For PVDF/PLLA/5%RCC, an AHR of 129.1% for the 50/50 blend corresponds to a 70.9% PVDF continuity, while AHRs much lower than 100% suggest poor PLLA continuity in the 70/30~90/10 blends (Figure 3A). Only the 60/40 and 65/35 blends of ~100% AHR can be co-continuous. The co-continuity interval of the PVDF/PLLA/5%RCC blend is therefore determined as 60/40~65/35. In the same way, the co-continuity intervals of PVDF/PLLA/10%RCC and PVDF/PLLA/20%RCC blends were examined as 30/70~65/35 and 10/90~60/40 respectively, based on the AHR values in Figure 3B,C.

### 3.2. Promoting Mechanism of RCC on PVDF/PLLA Co-Continuity

**Figure 4:**
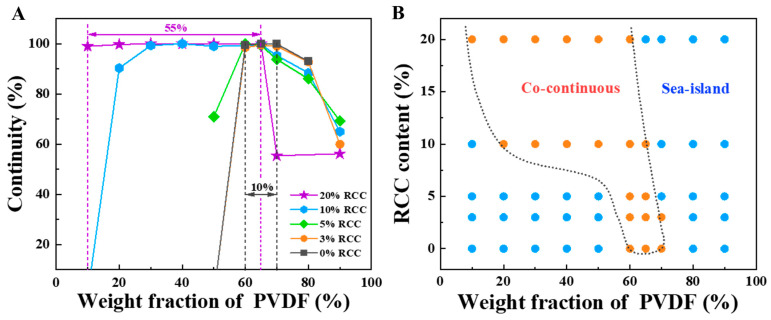
(**A**) The summary chart of the continuity of the PVDF/PLLA/RCC blends with various PVDF weight fractions. (**B**) The co-continuous phase diagram (black dotted line) of the PVDF/PLLA/RCC blends, depicted by the boundary between the co-continuous zone (orange points) and the sea-island zone (blue points).

**Figure 5:**
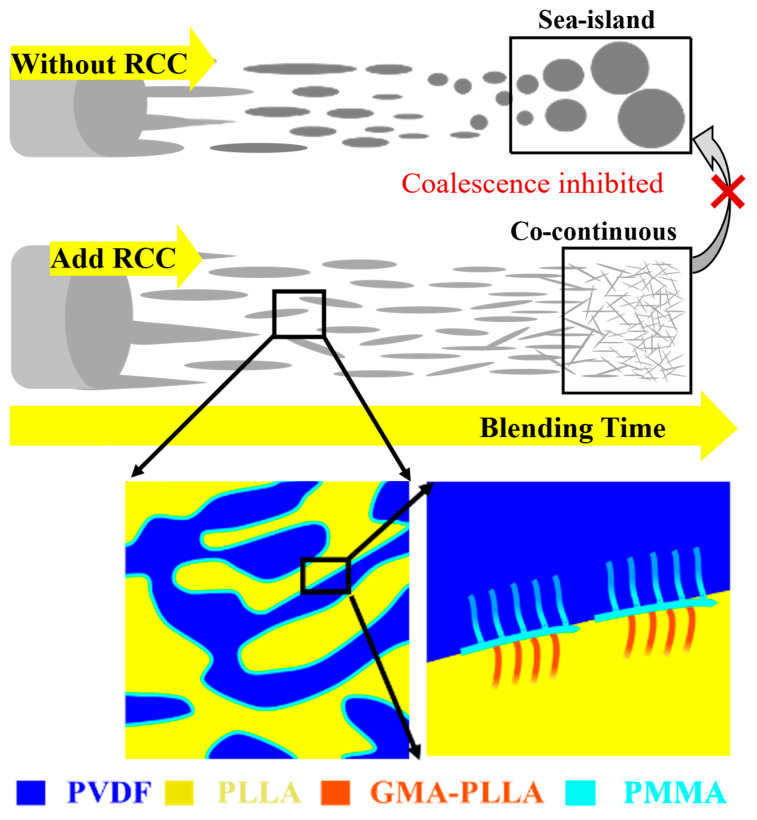
The evolution of PVDF and PLLA phases in the PVDF/PLLA blends without and with RCC, along with a partial enlarged view of the boundary between PVDF and PLLA phases in the RCC-compatibilized PVDF/PLLA blend system.

By gathering the continuity of the PVDF or PLLA phase in the PVDF/PLLA/RCC blends in Figure 4A, it can be clearly found that a significant alteration of co-continuity intervals occurs with increased RCC content. On the one hand, the interval of PVDF/PLLA/20%RCC, covering 55% PVDF fraction, is remarkably wider than that of the neat PVDF/PLLA blend (10%). On the other hand, the lower limit of the interval is extended from 60/40 (with 0% RCC) to 10/90 (with 20% RCC) while the upper boundary is slightly decreased from 70/30 to 60/40. As a result, a half-U-shaped phase diagram with an upward opening (depicted by a black dotted line in Figure 4B) is obtained, indicating a promoting effect of RCC on the PVDF/PLLA co-continuity. The underlying mechanism is thus illustrated in Figure 5 [31]. In the neat PVDF/PLLA blend, coalescence and recombination of the phases predominantly occur upon blending, leading to a sea-island structure with either PVDF or PLLA islands (depending on the PVDF/PLLA composites). When over 10% RCC is added into the blend, PVDF phase coalescence can be effectively inhibited by RCC, bringing about tiny PVDF droplets connected with each other and thus co-continuous structures. The conclusion also fits well with the results in a previous work [29], where PVDF islands in the PVDF/PLLA/RCC (30/70/20%) blend turned into smaller ones with the progress of blending, leading to a sub-100 nm co-continuous structure in the blend.

**Figure 6:**
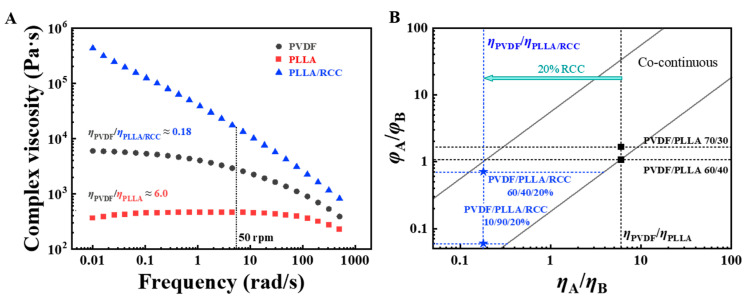
(**A**) The frequency-dependent complex viscosity of PVDF, PLLA, and PLLA/RCC. (**B**) The illustration diagram for the widening of the PVDF/PLLA co-continuity interval in the PVDF/PLLA/20%RCC blend compared with the neat PVDF/PLLA blend, with the PVDF/PLLA co-continuous region delimited by black solid lines.

Deeper reasons for the formation of the half-U-shaped phase boundary were further investigated by detecting the viscosity of neat PVDF, neat PLLA, and PLLA/20%RCC at 50 rpm rotation (signified as $\eta_{PVDF}$, $\eta_{PLLA}$, and $\eta_{PLLA/RCC}$ respectively) via frequency sweeping rheological measurement (Figure 6A). The PVDF/PLLA blends with ultrahigh PVDF compositions can be hardly co-continuous, due to the inclined PLLA phase coalescence, even at the PVDF/PLLA composition ratios (e.g., 80/20) close to their viscosity ratio. As a result, the composition ratio of the co-continuous PVDF/PLLA blend is limited to 60/40~70/30 according to the $\eta_{PVDF}/\eta_{PLLA}$. When the RCC (reactive with PLLA) is introduced into the blend, taking PVDF/PLLA/20%RCC as an example, the $\eta_{PLLA/RCC}$ can be elevated to ~15,500, much higher than the $\eta_{PVDF}$. Due to this thickening effect of RCC on PLLA, the ratio of $\eta_{PVDF}$ to $\eta_{PLLA(/RCC)}$ is significantly decreased from ~6.0 to ~0.18. It is well known that co-continuous structures are always formed in a blend with components A and B at a volume fraction ratio ($\varphi_{A}/\varphi_{B}$) basically equal to their viscosity ratio ($\eta_{A}/\eta_{B}$) [23,24], as delimited by gray solid lines in Figure 6B. Then, owing to the remarkable reduction in $\eta_{PVDF}$/$\eta_{PLLA(/RCC)}$ at an RCC addition of 20%, the co-continuity $\varphi_{PVDF}$/$\varphi_{PLLA(/RCC)}$ interval for the PVDF/PLLA/20%RCC blend is lowered accordingly. It is worth noting that even in the blend with over 70% PLLA, the existence of 20% RCC can effectively inhibit the PVDF phase coalescence and promote the formation of a co-continuous structure, in accordance with the illustration in Figure 5. That is, the synergism of high PLLA/RCC viscosity and inhibited PVDF phase coalescence is responsible for the promoting effect of RCC on the PVDF/PLLA co-continuity.

## 4. Conclusions

In this work, the co-continuous phase diagram of the PVDF/PLLA/RCC blend system was plotted using SEM morphology and acidolysis of PLLA, for illustrating the effect of RCC addition on the microphase structure for the blend system. Upon blending, the reaction occurs between terminal carboxyl groups in PLLA and epoxy groups in RCC, producing GMA-PLLA side chains located at the PVDF/PLLA interface. A half-U-shaped phase diagram with an upward opening was ultimately obtained, which revealed the promoting effect of RCC addition on the co-continuity formation in the blend. On the one hand, the significant decrease in viscosity ratio of PVDF to PLLA/RCC can lower the co-continuous PVDF/PLLA composition ratio. On the other hand, the PVDF phase coalescence in the PVDF/PLLA/RCC blends with high PLLA composition can be effectively inhibited due to the RCC addition. As a result, this work provides a strategy for evaluating the effect of RCs on co-continuity formation in immiscible polymer blends.

## Data Availability

The original contributions presented in this study are included in the article and Supplementary Material. Further inquiries can be directed to the corresponding authors.

## Supplementary Materials

The following supporting information can be downloaded at: https://www.mdpi.com/article/10.3390/polym18131586/s1, Figure S1: The GPC spectra of PLLA and PLLA/RCC; Figure S2: The SEM images of (A) solvent extracted and (B) acid hydrolyzed PVDF/PLLA 70/30 blend. Figure S2B is the partial enlargement image of the Figure 1E.
