# Supplementary material for: The Promoting Effect of Reactive Comb Compatibilizer on the Formation of Co-Continuous Structure in PVDF/PLLA Blends
Source: Polymers (Basel). 2026 Jun 26;18(13):1586. doi: 10.3390/polym18131586 (PMC13363733; doi:10.3390/polym18131586)
Supplement: Supplementary file 1 [file polymers-18-01586-s001.zip › polymers-4378711-supplementary.pdf]

Supporting Information

The Promoting Effect of Reactive Comb Compatibilizer on the Formation of Co-Continuous Structure in PVDF/PLLA Blends

Yufei Dong [1,2], Fei Li [1], Jiayao Wang [2], Yongjin Li [2], Guipeng Yu [1,*] and Jichun You [2,*]

[1] College of Chemistry and Chemical Engineering, Central South University, Changsha 410083, China
[2] College of Material, Chemistry and Chemical Engineering, Key Laboratory of Organosilicon Chemistry and Material Technology, Ministry of Education, Hangzhou Normal University, Hangzhou, 311121, Zhejiang, China
* Correspondence: you@hznu.edu.cn (J. You), gilbertyu@csu.edu.cn (G. Yu)

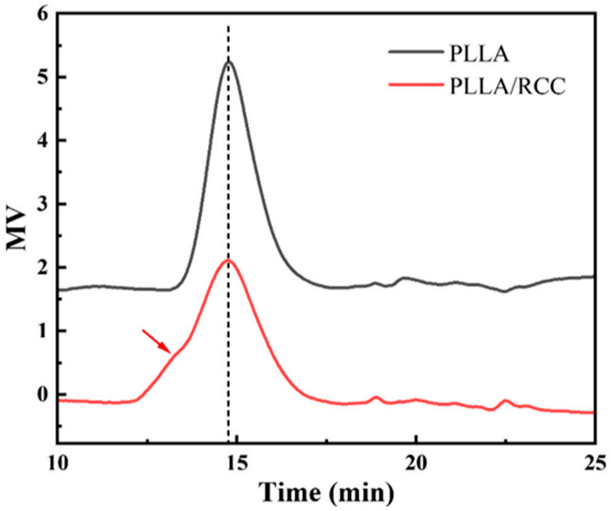

Figure S1. The GPC spectra of PLLA and PLLA/RCC.

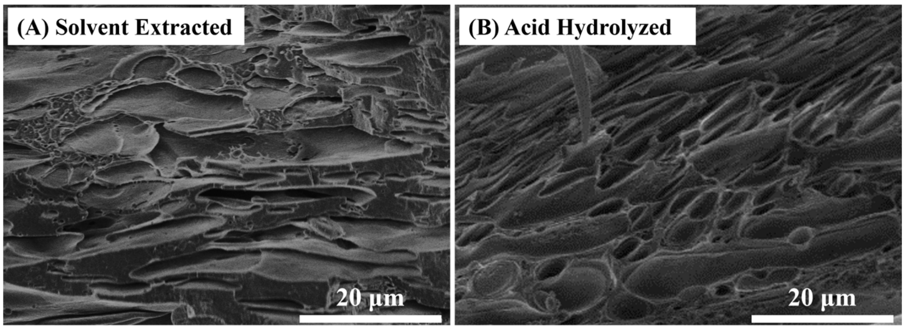

Figure S2. The SEM images of (A) solvent extracted and (B) acid hydrolyzed PVDF/PLLA 70/30 blend. Figure S2B is the partial enlargement image of the Figure 1E.
